# Supplementary material for: How does Indian news media report smokeless tobacco control? A content analysis of the gutka ban enforcement
Source: PLOS Glob Public Health. 2023 Mar 27;3(3):e0001724. doi: 10.1371/journal.pgph.0001724 (PMC10042338; doi:10.1371/journal.pgph.0001724)
Supplement: S1 Table — (PDF) [file pgph.0001724.s001.pdf]

S1 Table. News media reports included in the review.

Media file	Year	Headline	Location	Link
Files\\001	03-01-2018	Gutka worth Rs 1.4 crore seized	Telangana	https://timesofindia.indiatimes.com/city/hyderabad/gutka-worth-rs-1-4-crore-seized/articleshow/62346325.cms
Files\\002	12-09-2018	Gutka packets seized in Vijayawada	Andhra Pradesh	https://www.thehansindia.com/posts/index/Andhra-Pradesh/2018-09-12/Gutka-packets-seized-in-Vijayawada/411435
Files\\003	08-02-2018	Gutka-making unit seized in Hyderabad	Telangana	https://www.thehindu.com/news/cities/Hyderabad/gutka-making-unit-seized-in-hyderabad/article22683010.ece
Files\\004	26-03-2018	250 bags of gutka worth Rs 10 lakh seized from Dowleshwaram in Andhra Pradesh	Andhra Pradesh	http://www.newindianexpress.com/states/andhra-pradesh/2018/mar/26/250-bags-of-gutka-worth-rs-10-lakh-seized-from-dowleswaram-in-andhra-pradesh-1792492.html
Files\\005	15-03-2019	Gutka worth Rs 1 Cr seized in Hyderabad	Telangana	https://www.thehansindia.com/telangana/gutka-worth-rs-1-cr-seized-in-hyderabad-512258
Files\\006	20-12-2017	Gutka seizure a challenge	Telangana	https://www.deccanchronicle.com/nation/in-other-news/201217/gutka-seizure-a-challenge.html
Files\\007	26-03-2019	Gutka worth Rs 10 lakh seized in Srikakulam, one held	Andhra Pradesh	http://www.uniindia.com/gutka-worth-rs-10-lakh-seized-in-srikakulam-one-held/south/news/1541561.html
Files\\008	01-05-2019	VE officials seize gutka, mechines worth 25 lakh	Andhra Pradesh	https://www.thehansindia.com/andhra-pradesh/ve-officials-seize-gutka-machines-worth-25-lakh--525659
Files\\009	09-05-2019	Kadapa: Gutka packets worth Rs 6 lakh seized, two persons arrested	Andhra Pradesh	https://www.deccanchronicle.com/nation/crime/090519/kadapa-gutka-packets-worth-rs-6lakh-seized-two-persons-arrested.html
Files\\010	18-04-2019	236 kg ganja seized, two held in vijayawada (Gutka worth Rs 16 L seized)	Andhra Pradesh	http://www.newindianexpress.com/cities/vijayawada/2019/apr/18/236-kg-ganja-seized-2-held-1965805.html
Files\\011	13-10-2018	police arrested 10 member gang seized Rs 5.50 lakh gutka bags	Andhra Pradesh	https://www.thehansindia.com/posts/index/Andhra-Pradesh/2018-10-13/police-arrested-10-member-gang-seized-Rs-550lakh-Gutka-bags/424568
Files\\012	04-06-2019	Gutka worth Rs 10 L seized	Andhra Pradesh	http://www.newindianexpress.com/states/andhra-pradesh/2019/jun/04/gutka-worth-rs-10-l-seized-1985798.html
Files\\013	22-05-2018	Gutka packets worth 15 lakh seized, 9 held	Andhra Pradesh	https://www.thehansindia.com/posts/index/Andhra-Pradesh/2018-05-22/Gutka-packets-worth-15-lakh-seized-9-held/383107

Files\\014	01-10-2018	Gutka, ganja manufacturing gang held in kandukaur	Andhra Pradesh	https://www.thehansindia.com/posts/index/Andhra-Pradesh/2018-10-01/Gutkha-ganja-manufacturing-gang-held-in-Kandukur/416717
Files\\015	15-07-2017	Gutka seized in raids, illegal sale of chewing tobacco to be checked	Andhra Pradesh	http://www.newindianexpress.com/states/andhra-pradesh/2017/jul/15/gutka-seized-in-raids-illegal-sale-of--chewing-tobacco-to-be-checked-1628949.html
Files\\016	29-06-2018	Gutka sale continues unabated in Vijayawada	Andhra Pradesh	https://www.thehansindia.com/posts/index/Andhra-Pradesh/2018-06-29/Gutka-sale-continues-unabated-in-Vijayawada/393431
Files\\017	17-05-2018	Gutka packets worth 23 lakh seized	Andhra Pradesh	https://www.thehansindia.com/posts/index/Andhra-Pradesh/2018-05-17/Gutka-packets-worth-23-lakh-seized/381883
Files\\018	02-03-2018	Gutka worth 4 lakh seized	Andhra Pradesh	https://www.thehansindia.com/posts/index/Amaravati-Tab/2018-03-01/Gutkha-worth-4-lakh-seized/362583
Files\\019	24-08-2019	Gutka manufacturing unit unearthed in Prakasam	Andhra Pradesh	https://www.thehindu.com/news/cities/Vijayawada/gutkha-manufacturing-unit-unearthed-in-prakasam/article29237060.ece
Files\\020	25-09-2019	Fake cops held for robbing gutka vendors	Telangana	https://www.thehansindia.com/telangana/fake-cops-held-for-robbing-gutka-vendors-566922
Files\\021	28-12-2017	Gutka sale unabated in Nellore	Andhra Pradesh	https://www.thehansindia.com/posts/index/Andhra-Pradesh/2017-12-28/Gutka-sale-unabated-in-Nellore/348456
Files\\022	01-02-2017	Gutka sales cross100 cr a day!	Telangana	https://www.thehansindia.com/posts/index/Andhra-Pradesh/2017-02-01/Gutkha-sales-cross-100-cr-a-day/277355
Files\\023	30-08-2017	Arms haul; KMSS move; Leopard;Cattle seized; Green panel	Assam	https://www.telegraphindia.com/states/north-east/arms-haul-kmss-move-leopard-cattle-seized-green-panel/cid/1437892
Files\\024	24-07-2017	Golaghat police seize huge quantity of gutka and zarda	Assam	https://www.sentinelassam.com/news/golaghat-police-seize-huge-quantity-of-gutkha-and-zarda/
Files\\026	07-03-2019	Gutka worth Rs 1 lakh seized from a shop in Misarwadi	Maharashtra	https://timesofindia.indiatimes.com/city/aurangabad/gutka-worth-rs1l-seized-from-a-shop-in-misarwadi/articleshow/68293561.cms
Files\\027	12-08-2018	From pen drives to hammers: Massive raids across Bihar jail	Bihar	https://www.ndtv.com/patna-news/from-pen-drives-to-hammers-massive-raids-across-bihar-jails-1899090
Files\\028	07-12-2017	A truck with gutka seized, driver arrested	Bihar	https://www.livehindustan.com/bihar/chapra/story-gutka-seized-truck-seized-arrested-driver-1684254.html
Files\\029	22-05-2017	Gutka and pan masala truck seized in Chhapra	Bihar	https://www.livehindustan.com/news/patna/article1-gutkha-and-pan-masala-seized-truck-in-chhapra-836652.html

Files\\030	23-05-2016	40 lakh worth gutka truck seized, driver arrested	Bihar	https://www.jagran.com/bihar/kaimoor-40-million-including-chewing-tobacco-seized-truck-driver-arrested-14054355.html
Files\\031	09-10-2017	Hajipur: 22 lakh worth illegal gutka transportation seized by sonpur police	Bihar	https://patnalive.co.in/gutkha-loaded-truck-seized/
Files\\032	02-05-2019	Chhattisgarh police seize gutka worth Rs 50 lakh during raid in Durg	Chhattisgarh	http://www.newindianexpress.com/nation/2019/may/02/chhattisgarh-police-seize-gutka-worth-rs-50-lakh-during-raids-in-durg-1971841.html
Files\\033	06-11-2017	Gutka packets worth Rs. 6 lakh seized in rajnandgaon	Chhattisgarh	https://www.naidunia.com/chhattisgarh/rajnandgaon-seized-6-lakhs-rupees-tobacco-gutka-in-rajnandgaon-1387881
Files\\034	08-08-2016	4 people arrested with 1.5 crore worth gutka packets	Chhattisgarh	https://www.naidunia.com/chhattisgarh/bhilai-four-accused-arrested-with-tobacco-product-812231
Files\\036	19-09-2014	4 shops raided and 8 lakh worth gutka seized in the capital	Chhattisgarh	https://www.naidunia.com/chhattisgarh/raipur-gutkha-worth-rupees-8-lacs-seized-186392
Files\\037	30-04-2019	Police seized 95 bags of gutka worth 50 lakh, 4 accussed arrested.	Chhattisgarh	https://www.bhaskar.com/chhattisgarh/bhilaidurg/news/95-packet-banned-gutka-confiscated-in-bhilai-01534181.html
Files\\038	20-09-2018	Gariaband: Department of food civil supplies and consumer protection seized 266 gutka packets from a pan shop	Chhattisgarh	https://www.haribhoomi.com/chhattisgarh/garia-bandh-seized-266-packets-of-banned-gutka-department-of-food-and-drug-administration
Files\\039	27-08-2015	A godown raided and gutka worth Rs 10 lakh seized	Chhattisgarh	https://www.patrika.com/raipur-news/raipur-patrika-impact-warehouse-raid-seized-10-million-ghutka-1091023/
Files\\040	06-10-2015	50 lakh worth gutka seized in Birgaon	Chhattisgarh	https://www.patrika.com/raipur-news/raipur-birgaon-gutkha-of-50-lack-rupees-seized-1115464/
Files\\041	03-03-2013	A large quantity of gutka was seized in Bemetra, Raipur	Chhattisgarh	https://www.bhaskar.com/news/CHH-RAI-rajshree-gutkha-of-millions-seized-in-raipur-4196523-NOR.html
Files\\042	27-08-2016	Gutka worth 4 lakh transported from Delhi to Raipur and stored in a godown was seized.	Chhattisgarh	https://www.naidunia.com/chhattisgarh/raipur-4-millions-of-chewing-tobacco-seized-from-the-warehouse-was-brought-from-delhi-to-raipur-805387
Files\\044	30-04-2019	Gutka worth 50 lakh seized in Durga. Four arrested.	Chhattisgarh	https://thevoices.in/cache-of-gutkha-worth-lakshmi-seized-in-durg
Files\\045	29-11-2017	12 carton of gutka was seized in Bhatgaon, godown seeled.	Chhattisgarh	https://glibs.in/Crime/chhattisgarh-news-12-cartoon-gutka-seized-in-Bhatgaon-raipur-news-16230.html

Files\\046	04-06-2011	Panaji: Gutka pouches worth Rs 10 lac seized in Goa	Goa	https://www.daijiworld.com/news/newsDisplay.aspx?newsID=104342
Files\\047	05-06-2011	Gutka worth 10L seized at Ponda	Goa	https://timesofindia.indiatimes.com/city/goa/Gutka-worth-10L-seized-at-Ponda/articleshow/8729625.cms
Files\\048	24-01-2012	Truck carrying gutka worth Rs 3 lakh seized at Taleigao	Goa	https://timesofindia.indiatimes.com/city/goa/Truck-carrying-gutka-worth-Rs-3-lakh-seized-at-Taleigao/articleshow/11607552.cms
Files\\049	25-12-2018	Gutka freely on sale across Goa	Goa	https://navhindtimes.in/gutkha-freely-on-sale-across-goa/
Files\\052	29-06-2016	Foreign cigrettes, scented tobacco seized in Panaji	Goa	http://englishnews.thegoan.net/story.php?id=19682
Files\\054	18-01-2019	Owner of illegal gutka unit booked for Rs. 70 cr tax evasion	Gujarat	https://timesofindia.indiatimes.com/city/vadodara/owner-of-illegal-gutka-unit-booked-for-rs-70cr-tax-evasion/articleshow/67579345.cms
Files\\055	28-12-2015	post-ban, gutkas of 50,000 firms seized in three years	Gujarat	https://indianexpress.com/article/cities/ahmedabad/post-ban-gutkas-of-50000-firms-seized-in-three-years/
Files\\059	08-08-2019	Two held for making gutka	Gujarat	https://timesofindia.indiatimes.com/city/ahmedabad/two-held-for-making-gutka/articleshow/70578364.cms
Files\\060	11-08-2017	DRI arrests two; seizes counterfeit gutkha to be sent to Kuwait via Gujarat	Gujarat	https://www.deshgujarat.com/2017/08/11/
Files\\063	12-09-2012	Widespread raids to spit out gutka menace	Gujarat	https://timesofindia.indiatimes.com/city/ahmedabad/Widespread-raids-to-spit-out-gutka-menace/articleshow/16360041.cms
Files\\064	26-08-2012	FDA tightens noose around gutka shops	Chandigarh	https://www.hindustantimes.com/chandigarh/fda-tightens-noose-around-gutkha-shops/story-c7AkMocq0XK2RXkVFa2EwJ.html
Files\\065	08-09-2012	7.5 quintals gutka seized in raid	Haryana	https://www.tribuneindia.com/2012/20120908/haryana.htm#6
Files\\067	19-07-2019	large quantity of banned tobacco products seized in Shimla city	Himachal Pradesh	https://himachalwatcher.com/2019/07/19/large-quantity-of-banned-tobacco-products-seized-in-shimla-city/
Files\\068	02-06-2017	DGCEI busts illegal gutka unit, detects Rs 400 crore tax evasion	Karnataka	https://economictimes.indiatimes.com/news/politics-and-nation/dgcei-busts-illegal-gutkha-unit-detects-rs-400-crore-tax-evasion/articleshow/58963203.cms?from=mdr
Files\\069	26-05-2019	Four wheelers, including 27,000 cash, 3 bags gutka seized	Madhya Pradesh	https://www.patrika.com/tikamgarh-news/four-wheelers-including-27-thousand-cash-three-baga-gutka-seized-4621681/

Files\\070	05-05-2015	Through Rajashtan, the smuggling of gutka and pan masala started in the region, 75 lakh worth gutka and pan masala was seized.	Madhya Pradesh	https://hindi.news18.com/news/madhya-pradesh/gwalior-rs-75-lakh-gutkha-and-pan-masala-seized-709648.html
Files\\071	14-03-2019	10 lakh gutka seized	Maharashtra	http://www.lokmat.com/ratnagiri/10-lakhs-gutka-seized/
Files\\072	31-03-2019	Gutka worth Rs 51.83 lakh seized in Maharashtra, 3 arrested	Maharashtra	https://www.business-standard.com/article/pti-stories/gutkha-worth-rs-51-83-lakh-seized-in-maha-3-arrested-119033100099_1.html
Files\\073	20-07-2018	Gutka , illicit booze worth Rs 9.9 lakh seized	Maharashtra	https://www.asianage.com/metros/mumbai/200718/gutka-illicit-booze-worth-rs-99lakh-seized.html
Files\\074	28-03-2019	Gutka worth Rs 1.77 crore seized in Maharshtra; 8 arrested	Maharashtra	https://www.business-standard.com/article/pti-stories/gutkha-worth-rs-1-77-crore-seized-in-maharashtra-8-arrested-119032800849_1.html
Files\\075	17-05-2019	Gutka worth 6.5 lakh seized by police in Ulhasnagar	Maharashtra	https://www.esakal.com/mumbai/gutka-seized-police-rs-6-and-half-lakhs-ulhasnagar-189367
Files\\076	04-07-2015	Maharshtra mulls making sale of gutka non-bailable offence	Maharashtra	https://www.downtoearth.org.in/news/maharshtra-mulls-making-sale-of-gutkha-nonbailable-offence-49167
Files\\077	30-10-2018	Gutka worth Rs 44 lakh seized, two arrested in Maharashtra	Maharashtra	http://www.newindianexpress.com/nation/2018/oct/30/gutka-worth-rs-44-lakh-seized-two-arrested-in-maharashtra-1891928.html
Files\\078	10-07-2018	Gutka worth 1.5 crore was seized in two months	Maharashtra	https://maharashtratimes.indiatimes.com/maharashtra/thane-kokan-news/thane/gutkha-of-one-and-half-crores-seized-in-two-months/articleshow/64923058.cms
Files\\079	04-10-2019	Lakhs worth of gutka seized in Gandhidham	Gujarat	https://indianexpress.com/article/cities/ahmedabad/lakhs-worth-of-gutka-seized-in-gandhidham/
Files\\082	02-05-2019	Bombay HC refuses to stay FDA diktat on transportation of banned gutka	Maharashtra	https://timesofindia.indiatimes.com/city/mumbai/hc-refuses-to-stay-fda-diktat-on-transportation-of-banned-gutka/articleshow/69136797.cms
Files\\083	16-12-2018	FIR against six in Rs 28 lakh gutka seizure case	Maharashtra	https://timesofindia.indiatimes.com/city/mumbai/fir-against-six-in-rs-28-lakh-gutkha-seizure-case/articleshow/67110960.cms
Files\\084	04-04-2019	Gutka worth Rs 8.5 lakh seized in Hatkanangle	Maharashtra	https://timesofindia.indiatimes.com/city/kolhapur/gutka-worth-rs-8-5-lakh-seized-in-hatkanangle/articleshow/68711989.cms
Files\\085	21-08-2018	Gutka worth Rs. 1 crore seized near Mumbai, 2 held	Maharashtra	https://timesofindia.indiatimes.com/city/thane/gutkha-worth-rs-1-crore-seized-near-mumbai-2-held/articleshow/65492943.cms
Files\\086	30-10-2018	Gutka worth Rs 12 lakh seized at Anewadi toll plaza, 2 held	Maharashtra	https://timesofindia.indiatimes.com/city/kolhapur/gutka-worth-rs-12-lakh-seized-at-anewadi-toll-plaza-2-held/articleshow/66421308.cms

Files\\087	23-03-2019	Banned gutka worth Rs 7.41 crore destroyed	Maharashtra	https://timesofindia.indiatimes.com/city/pune/banned-gutka-worth-rs-7-41cr-destroyed/articleshow/68529548.cms
Files\\089	01-11-2018	Sale of gutka becomes non-bailable offence in Maharashtra	Maharashtra	https://www.hindustantimes.com/mumbai-news/sale-of-gutkha-becomes-non-bailable-offence-in-maharashtra/story-i75yeCcNQ54t2CLMC7wFhN.html
Files\\091	19-04-2019	2 arrested, cash, gutka confiscated	Maharashtra	https://timesofindia.indiatimes.com/city/kolhapur/2-arrested-cash-gutka-confiscated/articleshow/68952947.cms
Files\\092	18-05-2019	Over 20 lakh rupees worth of gutka seized	Maharashtra	http://www.lokmat.com/akola/over-20-lakh-rupees-worth-gutka-seized/
Files\\094	07-10-2019	Maharashtra to make sale of gutka non-bailable offence	Maharashtra	https://indianexpress.com/article/india/maharashtra-to-make-sale-of-gutka-non-bailable-offence-5089275/
Files\\095	11-03-2019	Two crore gutka seized on the Pune-Bengaluru highway	Maharashtra	https://www.esakal.com/paschim-maharashtra/two-crore-gutka-seized-pune-bengaluru-highway-176059
Files\\096	04-05-2019	12 lakhs of banned gutka seized	Maharashtra	http://www.lokmat.com/akola/12-lakhs-banned-gutka-seized/
Files\\098	19-07-2018	FDA seals six pan shops for selling gutka	Maharashtra	https://timesofindia.indiatimes.com/city/nagpur/fda-seals-6-pan-shops-for-selling-gutkha/articleshow/65045633.cms
Files\\099	07-10-2019	Police seize gutka worth Rs 12 lakh from tempo near Mumbai	Maharashtra	https://indianexpress.com/article/cities/mumbai/police-seize-gutkha-worth-rs-12-lakh-from-tempo-near-mumbai-5073877/
Files\\100	16-10-2012	FDA wants Gutka owners to pay for seized goods destruction	Maharashtra	https://www.moneylife.in/article/fda-wants-gutka-owners-to-pay-for-seized-goods-destruction/29104.html
Files\\101	24-11-2018	Gutka worth Rs 4.52 lakh seized in Mumbra	Maharashtra	https://timesofindia.indiatimes.com/city/thane/gutka-worth-rs-4-52-lakh-seized-in-mumbra/articleshow/66785120.cms
Files\\102	05-03-2017	Gutka truck seized	Maharashtra	https://www.lokmat.com/aurangabad/gutkhas-truck-seized/
Files\\103	26-05-2019	Gutka worth Rs 70 lakh seized from train in Mumbai	Maharashtra	https://www.business-standard.com/article/pti-stories/gutkha-worth-rs-70-lakh-seized-from-train-in-mumbai-119052600426_1.html
Files\\104	15-08-2018	Gutka worth Rs 11.56 lakh seized in Thane	Maharashtra	http://www.uniindia.com/gutka-worth-rs-11-56-seized-in-thane/states/news/1321033.html
Files\\105	22-01-2019	Odisha: Govt bus stashed with gutka sacks seized; 2 held	Odisha	https://odishasuntimes.com/odisha-govt-bus-stashed-with-gutkha-sacks-seized-2-held/
Files\\106	22-01-2019	50 sacks of gutka seized from bus in Bhubaneshwar	Odisha	http://www.odishabytes.com/50-sacks-of-gutka-seized-from-bus-in-bhubaneswar/
Files\\108	04-06-2017	Jails across Odisha raided	Nagaland	http://www.easternmirrornagaland.com/nation-news-in-brief-39/

Files\\109	01-06-2017	70 lakh worth gutka seized in alwar	Rajasthan	https://www.khaskhabar.com/local/rajasthan/alwar-news/news-seized-gutka-of-70-lakh-rupees-in-alwar-news-hindi-1-221264-KKN.html
Files\\110	02-10-2019	Rajashtan bans certain categories of pan masala	Rajasthan	https://www.thehindu.com/news/national/other-states/rajasthan-bans-certain-categories-of-pan-masala/article29573900.ece
Files\\111	13-09-2013	6 more tonnes of gutka seized in Chennai	Tamil Nadu	https://www.thehindu.com/news/cities/chennai/6-more-tonnes-of-gutka-seized-in-chennai/article5121110.ece
Files\\112	08-05-2018	In 3rd bust on train from Delhi, 289 kg gutka worth Rs 6 lakh seized at city station	Tamil Nadu	https://timesofindia.indiatimes.com/city/chennai/in-3rd-bust-on-train-from-delhi-287kg-gutka-worth-rs-6l-seized-at-city-station/articleshow/64070949.cms
Files\\113	14-08-2013	Gutka-carrying lorry held	Tamil Nadu	https://www.thehindu.com/news/cities/chennai/gutkacarrying-lorry-held/article5019835.ece
Files\\114	28-06-2018	TN-Seizure-Gutka	Tamil Nadu	https://www.indiatoday.in/pti-feed/story/tn-seizure-gutka-1272327-2018-06-28
Files\\115	12-11-2018	300 kg of gutka seized in Coimbatore	Tamil Nadu	https://timesofindia.indiatimes.com/city/coimbatore/300kg-of-gutka-seized-in-coimbatore/articleshow/66594194.cms
Files\\116	26-05-2018	840 kilogram gutka seized from two parked trucks	Tamil Nadu	https://timesofindia.indiatimes.com/city/coimbatore/840kg-gutka-seized-from-2-parked-trucks/articleshow/64325611.cms
Files\\117	12-08-2018	Banned gutka products worth rs 22.5 lakh seized	Tamil Nadu	https://www.thehindu.com/news/cities/Coimbatore/banned-gutkha-products-worth-225-lakh-seized/article24669471.ece
Files\\118	16-11-2018	450kg of gutka seized by FSSAI	Tamil Nadu	https://www.dtnext.in/News/TamilNadu/2018/11/16044940/1095816/450-kg-of-gutka-seized-by-FSSAI.vpf
Files\\119	09-11-2018	Over 6 tonnes of gutka seized at Poonamallee, 5 arrested	Tamil Nadu	https://newstodaynet.com/index.php/2018/11/09/over-6-tonnes-of-gutkha-seized-at-poonamallee-5-arrested/
Files\\121	17-05-2019	Two tonnes of gutka and tobacco seized	Tamil Nadu	https://www.thehindu.com/news/cities/Tiruchirapalli/two-tonnes-of-gutkha-and-tobacco-seized/article27164473.ece
Files\\122	03-05-2019	Rs. 50 lakh worth gutka seized in Vellore, five persons held	Tamil Nadu	http://www.newindianexpress.com/states/tamil-nadu/2019/may/03/rs-50-lakh-worth-gutka-seized-in-vellore-five-persons-held-1972296.html
Files\\123	18-05-2019	2 tonnes of gutka products seized	Tamil Nadu	https://timesofindia.indiatimes.com/city/trichy/2-tonnes-of-gutka-products-seized/articleshow/69380828.cms
Files\\124	02-05-2019	Police seize 575 kg of banned gutka, arrest 3	Tamil Nadu	https://timesofindia.indiatimes.com/city/trichy/police-seize-575kg-of-banned-gutka-arrest-3/articleshow/69136272.cms

Files\\125	02-05-2019	Conflicting laws help gutka sale thrive in TN	Tamil Nadu	https://timesofindia.indiatimes.com/city/trichy/conflicting-laws-help-gutka-sale-thrive-in-tn/articleshow/69136298.cms
Files\\126	03-05-2019	Rs 8 lakh worth banned tobacco products seized in Ariyalur	Tamil Nadu	https://timesofindia.indiatimes.com/city/trichy/rs-8-lakh-worth-banned-tobacco-products-seized-in-ariyalur/articleshow/69164522.cms
Files\\127	13-04-2019	Rs 7.5 lakh worth gutka products seized from truck	Tamil Nadu	https://timesofindia.indiatimes.com/city/trichy/rs-7-5l-worth-gutka-products-seized-from-truck/articleshow/68857723.cms
Files\\128	26-09-2018	2,350 kg of gutka seized in Coimbatore	Tamil Nadu	https://timesofindia.indiatimes.com/city/coimbatore/2350kg-of-gutka-seized-in-coimbatore/articleshow/65967474.cms
Files\\129	22-09-2018	Chennai: 5 tonnes of gutka, 3 trucks seized; 2 held	Tamil Nadu	https://www.deccanchronicle.com/nation/crime/220918/chennai-5-tonnes-of-gutka-3-trucks-seized-2-held.html
Files\\130	10-11-2018	Police seize 6.5 tonnes of gutka in chennai	Tamil Nadu	https://timesofindia.indiatimes.com/city/chennai/police-seize-6-5-tonnes-of-gutka-in-chennai/articleshow/66565076.cms
Files\\131	17-09-2018	250kg of gutka products seized in Trichy	Tamil Nadu	https://timesofindia.indiatimes.com/city/trichy/250kg-of-gutka-products-seized-in-trichy/articleshow/65845320.cms
Files\\132	22-01-2019	4.5 tonnes of banned tobacco products seized in Trichy	Tamil Nadu	https://timesofindia.indiatimes.com/city/trichy/4-5-tonnes-of-banned-tobacco-products-seized-in-trichy/articleshow/67632597.cms
Files\\133	02-10-2018	Chennai: 1.5 tonnes of gutka products seized from car near Poonamalle	Tamil Nadu	https://www.deccanchronicle.com/nation/crime/021018/15-tonne-gutka-products-seized-from-car-near-poonamallee.html
Files\\134	28-08-2018	chennai police seize banned gutka, tobacco products; 7 held	Tamil Nadu	https://www.deccanchronicle.com/nation/crime/280818/chennai-police-seize-banned-gutka-tobacco-products-7-held.html
Files\\135	23-09-2018	police seize 8 tonnes of gutka in raids, 8 held	Tamil Nadu	https://timesofindia.indiatimes.com/city/chennai/police-seize-8-tonnes-of-gutka-in-raids-8-held/articleshow/65919884.cms
Files\\136	18-09-2018	200 kg of gutka products seized in Erode	Tamil Nadu	https://timesofindia.indiatimes.com/city/coimbatore/200kg-of-gutka-products-seized-in-erode/articleshow/65848964.cms
Files\\137	24-03-2019	Tobacco products seized	Tamil Nadu	https://www.thehindu.com/news/cities/Coimbatore/tobacco-products-seized/article26622188.ece
Files\\138	29-09-2018	3600 kg gutka products seized from a godown near Manachanallur	Tamil Nadu	https://timesofindia.indiatimes.com/city/trichy/3600kg-gutka-products-seized-from-a-godown-near-manachanallur/articleshow/66007161.cms
Files\\139	05-10-2018	Premises of top GST, Central Excise personnel searched in gutka scam	Tamil Nadu	http://www.newindianexpress.com/states/tamil-nadu/2018/oct/05/premises-of-top-gst-central-excise-personnel-searched-in-gutkha-scam-1881356.html

Files\\140	25-12-2018	Gutka seized from Bengaluru train at Perambur railway station	Tamil Nadu	https://newstodaynet.com/index.php/2018/12/25/gutkha-seized-from-bengaluru-train-at-perambur-railway-station/
Files\\141	26-08-2018	Banned gutka sale goes on unabated	Tamil Nadu	https://timesofindia.indiatimes.com/city/chennai/banned-gutka-sale-goes-on-unabated/articleshow/63917598.cms
Files\\142	04-08-2018	Banned gutka products worth Rs. 3.5 lakh seized, two detained	Tamil Nadu	https://timesofindia.indiatimes.com/city/salem/banned-gutka-products-worth-rs-3-5-lakh-seized-two-detained/articleshow/65274590.cms
Files\\143	03-05-2018	FIR registered in gutka seizures from train	Tamil Nadu	https://timesofindia.indiatimes.com/city/chennai/fir-registered-in-gutka-seizures-from-train/articleshow/64007327.cms
Files\\144	28-04-2018	Coimbatore police seize 3.24 lakh sachets of gutka	Tamil Nadu	https://timesofindia.indiatimes.com/city/coimbatore/coimbatore-police-seize-3-24-lakh-sachets-of-gutka/articleshow/63949288.cms
Files\\145	15-08-2018	Chennai police seize Rs 50,000 worth gutka, two held	Tamil Nadu	https://www.deccanchronicle.com/nation/crime/150818/chennai-police-seize-rs-50000-worth-gutka-two-held.html
Files\\146	11-05-2019	Banned gutka worth Rs 3 L seized in Erode	Tamil Nadu	https://timesofindia.indiatimes.com/city/erode/banned-gutka-worth-rs-3l-seized-in-erode/articleshow/68349794.cms
Files\\147	27-06-2018	225 kg of banned gutka products seized	Tamil Nadu	https://timesofindia.indiatimes.com/city/erode/225kg-of-banned-gukta-products-seized/articleshow/64754660.cms
Files\\148	21-02-2019	Chennai: Gutka smugglers held with 5 tonnes of banned product	Tamil Nadu	https://www.timesnownews.com/mirror-now/crime/article/chennai-gutka-smugglers-held-with-5-tonnes-of-banned-product/369927
Files\\149	04-06-2019	Chennai: Man who tipped off cops assaulted by gutka sellers	Tamil Nadu	http://www.newindianexpress.com/cities/chennai/2019/jun/04/chennai-man-who-tipped-off-cops-assaulted-by-gutka-sellers-1985593.html
Files\\150	10-05-2019	240 kg gutka smuggled out via Central station parcel office	Tamil Nadu	https://timesofindia.indiatimes.com/city/chennai/240kg-gutka-smuggled-out-via-central-station-parcel-office/articleshow/69258496.cms
Files\\151	04-05-2019	chennai: Rs 50 lakh worth gutka seized, five arrested	Tamil Nadu	https://www.deccanchronicle.com/nation/current-affairs/040519/chennai-rs-50-lakh-worth-gutka-seized-five-arrested.html
Files\\152	12-03-2018	Gutka: Tamil Nadu oppositioin flags crores, fine's just Rs 55,000	Tamil Nadu	https://timesofindia.indiatimes.com/city/chennai/gutka-tamil-nadu-opposition-flags-crores-fines-just-55000/articleshow/63261915.cms
Files\\153	27-08-2018	chennai:2 held for selling gutka, 128 kg of banned products seized	Tamil Nadu	https://www.deccanchronicle.com/nation/crime/270818/chennai-2-held-for-selling-gutka-128-kg-of-banned-products-seized.html
Files\\154	18-09-2018	Over 200 kgs of banned tobacco products seized	Tamil Nadu	https://www.covaipost.com/coimbatore/over-200-kgs-of-banned-tobacco-products-seized/

Files\\155	22-11-2018	five ganja peddlers held in chennai	Tamil Nadu	https://www.deccanchronicle.com/nation/crime/221118/five-ganja-peddlers-held-in-chennai.html
Files\\156	10-03-2019	Restricted tobacco product of Rs 3 lakh was seized from a warehouse in Erode district of Tamil Nadu	Tamil Nadu	https://www.gnsnews.co.in/restricted-tobacco-product-of-rs-3-lakh-was-seized-from-a-warehouse-in-erode-district-of-tamil-nadu/
Files\\158	28-06-2013	1,000 kg of gutka, pan masala seized	Tamil Nadu	https://www.thehindu.com/news/cities/chennai/1000-kg-of-gutka-pan-masala-seized/article4857700.ece
Files\\159	26-05-2018	2 held with 850 kg gutka products	Tamil Nadu	https://www.deccanchronicle.com/nation/crime/260518/2-held-with-850-kg-gutka-products.html
Files\\160	18-05-2019	Rs 7.9 lakh worth gutka seized in Karimnagar	Telangana	https://telanganatoday.com/rs-7-9-lakh-worth-gutka-seized-in-karimnagar
Files\\163	28-05-2019	Cops seize 15 lakh worth gutka packets in Kothagudem	Telangana	https://www.thehansindia.com/telangana/cops-seize-15-lakh-worth-gutka-packets-in-kothagudem-532954
Files\\164	20-05-2019	Banned gutka worth Rs 21 lakh seized in Nirmal	Telangana	https://telanganatoday.com/banned-gutka-worth-rs-21-lakh-seized-in-nirmal
Files\\165	25-05-2019	Task force busts gutka racket	Telangana	https://www.thehansindia.com/telangana/task-force-busts-gutka-racket-532254
Files\\166	07-05-2019	Four held for smuggling gutka	Telangana	https://www.thehindu.com/news/cities/Hyderabad/four-held-for-smuggling-gutka/article27051938.ece
Files\\167	24-05-2019	Gutka worth Rs 1.5 lakh seized : 2 held	Telangana	http://www.uniindia.com/gutka-worth-rs-1-5-lakhs-seized-2-held/south/news/1610057.html
Files\\168	07-05-2019	Hyderabad: Rs 1.43 crore worth tobacco seized, four arrested	Telangana	http://www.newindianexpress.com/cities/hyderabad/2019/may/07/hyderabad-rs-143-crore-worth-tobacco-seized-four-arrested-1973539.html
Files\\169	09-05-2019	gang trading gutka products busted in Karimnagar	Telangana	https://telanganatoday.com/gang-trading-gutkha-products-busted-in-karimnagar
Files\\170	01-10-2018	Hyderabad: Police seize gutka worth Rs 13 lakh, five held	Telangana	https://www.deccanchronicle.com/nation/crime/011018/hyderabad-police-seize-gutka-worth-rs-13-lakh-five-held.html
Files\\171	16-03-2019	Gutka smuggling gangsters arrested, 1.2 cr products seized	Telangana	https://www.thehansindia.com/news/crime/gutka-smuggling-gangsters-arrested-12-cr-products-seized-512567
Files\\172	15-03-2019	Hyderabad: Gutka worth Rs 1.20 crore seized, six people held	Telangana	https://timesofindia.indiatimes.com/city/hyderabad/hyderabad-gutka-worth-rs-1-20-crore-seized-six-people-held/articleshow/68427438.cms

Files\\173	16-01-2019	Gutka worth Rs 27 lakh seized in Nirmal	Telangana	https://telanganatoday.com/gutka-worth-rs-27-lakh-seized-in-nirmal
Files\\174	12-02-2019	Rs 10.5 lakh gutka seized in Meeerpet PS limit	Telangana	https://www.thehansindia.com/posts/index/Latest-News/2019-02-12/Rs-105-lakh-gutka-seized-in-Meerpet-PS-limits/493246
Files\\175	17-09-2018	Gutka worth 15 lakh seized, 8 held in Hyderabad	Telangana	https://www.thehansindia.com/posts/index/Telangana/2018-09-17/Gutka-worth-15-lakh-seized-8-held-in-Hyderabad/412208
Files\\176	15-03-2019	Hyderabad: Rs 1 cr worth gutka products seized	Telangana	https://telanganatoday.com/hyderabad-rs-1-cr-worth-gutkha-products-seized
Files\\177	15-01-2019	Gutka sale on rise in north Andhra	Andhra Pradesh	https://www.thehansindia.com/posts/index/Andhra-Pradesh/2019-01-15/Gutka-sale-on-rise-in-north-Andhra/475588
Files\\179	01-02-2019	Gutka worth Rs 3.25 lakh seized	Telangana	https://www.thehansindia.com/posts/index/Latest-News/2019-02-01/Gutka-worth-Rs-325-lakh-seized/486136
Files\\180	23-03-2019	2 gutka smugglers held	Telangana	https://www.thehansindia.com/telangana/2-gutka-smugglers-held-514538
Files\\182	22-02-2019	Traders booked for sale of gutka packets	Telangana	https://www.thehansindia.com/posts/index/Telangana/2019-02-22/Traders-booked-for-sale-of-gutka-packets/499456
Files\\183	24-01-2019	Kirana shop owner held for selling gutka	Telangana	https://www.thehansindia.com/posts/index/Crime/2019-01-24/Kirana-shop-owner-held-for-selling-gutka/480948
Files\\184	02-04-2019	Hyderabad: Rs 24 lakh, 29 kg silver articles seized	Telangana	https://www.deccanchronicle.com/nation/current-affairs/020419/hyderabad-rs-24-lakh-29-kg-silver-articles-seized.html
Files\\185	20-09-2018	gutka, amber worth Rs 26 lakh seized, 6 held in Warangal	Telangana	https://telanganatoday.com/gutkha-amber-worth-rs-26-lakh-seized-6-held-in-warangal
Files\\186	22-03-2018	Gutka packets worth Rs 50 lakh seized	Telangana	https://www.thehindu.com/news/cities/Hyderabad/gutka-packets-worth-50-lakh-seized/article23315170.ece
Files\\187	21-08-2018	six gutka traders held	Telangana	https://www.thehansindia.com/posts/index/Telangana/2018-09-21/Six-gutkha-traders-held/413174
Files\\188	07-05-2019	Gutka and tobacco products worth 1.5 crore were seized from near Secunderabad railway station, four accused arrested	Telangana	https://hindi.sakshi.com/crime/2019/05/07/4-accused-arrested-transporting-illegal-gutka-in-hyderabad
Files\\189	03-09-2018	Hyderabad: two held for selling ganja, gutka	Telangana	https://www.deccanchronicle.com/nation/crime/030918/hyderabad-two-held-for-selling-ganja-gutka.html

Files\\190	28-06-2018	Hyderabad: five arrested for illegal transport of gutka	Telangana	https://www.deccanchronicle.com/nation/crime/280618/hyderabad-five-arrested-for-illegal-transport-of-gutka.html
Files\\191	29-05-2018	Gutka worth Rs 57.35 lakh seized in Hanamkonda	Telangana	https://www.thehansindia.com/posts/index/Warangal-Tab/2018-05-29/Gutka-worth-Rs-5735-lakh-seized-in-Hanamkonda/384899
Files\\192	10-04-2018	Huge cache of gutka seized, 6 held in Miryalaguda	Telangana	https://telanganatoday.com/huge-cache-of-gutka-seized-6-held-in-miryalaguda
Files\\193	20-05-2018	Gutka worth rs 12 lakh seized in Suryapet	Telangana	https://telanganatoday.com/gutkha-worth-rs-12-lakh-seized-in-suryapet
Files\\194	20-11-2018	In poll-bound TS, just cash and booze won't do	Telangana	https://www.thehindu.com/news/national/telangana/in-poll-bound-ts-just-cash-and-booze-wont-do/article25542080.ece
Files\\195	26-07-2014	150 bags of gutka, khaini seized	Andhra Pradesh	https://timesofindia.indiatimes.com/city/visakhapatnam/150-bags-of-gutka-khaini-seized/articleshow/39004192.cms
Files\\197	19-05-2018	Arunachal Pradesh: Banned tobacco product seized	Arunachal Pradesh	https://www.thenortheasttoday.com/archive/arunachal-pradesh-banned-tobacco-product-seized/
Files\\198	03-11-2019	One held with Rs 25 lakh gutka in raid	Maharashtra	https://timesofindia.indiatimes.com/city/mumbai/one-held-with-rs-25-lakh-gutka-in-raid/articleshow/71874855.cms
Files\\199	03-08-2012	Gutka worth Rs 1.4 crore seized in Maharashtra after ban	Maharashtra	https://www.business-standard.com/article/pti-stories/gutka-worth-rs-1-4-crore-seized-in-maharashtra-after-ban-112080300546_1.html
Files\\200	20-09-2019	Mumbai: Two held with Rs 34 lakh cigarettes, gutka	Maharashtra	https://timesofindia.indiatimes.com/city/mumbai/mumbai-two-held-with-rs-34-lakh-cigarettes-gutka/articleshow/71212164.cms
Files\\201	03-09-2019	Gutka worth Rs 6L seized, 1 arrested	Maharashtra	https://timesofindia.indiatimes.com/city/nashik/gutka-worth-rs-6l-seized-1-arrested/articleshow/70951093.cms
Files\\202	28-11-2018	Gutka, paan masala worth Rs 22L seized	Maharashtra	https://timesofindia.indiatimes.com/city/pune/gutka-paan-masala-worth-rs-22l-seized/articleshow/66835936.cms
Files\\203	21-07-2019	Vehicles carrying gutka will lose their licence now	Maharashtra	https://timesofindia.indiatimes.com/city/mumbai/vehicles-carrying-gutka-will-lose-their-licence-now/articleshow/70312730.cms
Files\\204	20-11-2019	Revenue dept officials begin crackdown on banned gutka	Tamil Nadu	https://timesofindia.indiatimes.com/city/coimbatore/revenue-dept-officials-begin-crackdown-on-banned-gutka/articleshow/72133728.cms
Files\\205	14-03-2019	950kg banned gutka products seized in 5 days	Tamil Nadu	https://timesofindia.indiatimes.com/city/coimbatore/950kg-banned-gutka-products-seized-in-5-days/articleshow/68400143.cms
Files\\206	06-09-2019	lllegal sale of gutka busted in Karur, 4 held	Tamil Nadu	https://www.newindianexpress.com/states/tamil-nadu/2019/sep/06/lllegal-sale-of-gutka-busted-in-karur-4-held-2029512.html

Files\\207	28-04-2018	648 kg of gutka in three lakh packets seized in Tamil Nadu, three held	Tamil Nadu	https://www.newindianexpress.com/states/tamil-nadu/2018/apr/29/648-kg-of-gutka-in-three-lakh-packets-seized-in-tamil-nadu-three-held-1807751.html
Files\\208	03-05-2018	FSSAI, police vow to continue crackdown against illegal units	Tamil Nadu	https://timesofindia.indiatimes.com/city/coimbatore/fssai-police-vow-to-continue-crackdown-against-illegal-units/articleshow/64007205.cms
Files\\209	10-10-2019	Adilabad: Gutka worth 50 lakh seized	Telangana	https://www.thehansindia.com/telangana/adilabad-gutka-worth-50-lakh-seized-571339
Files\\210	26-07-2018	Police raids on shops; 40 bags of gutka seized	Telangana	https://www.thehansindia.com/posts/index/Telangana/2018-07-26/Police-raids-on-shops-40-bags-of-gutka-seized/401122
Files\\211	17-08-2019	Rs 3.25 lakh worth gutka seized in Karimnagar	Telangana	https://telanganatoday.com/rs-3-25-lakh-worth-gutka-seized-karimnagar
Files\\212	14-03-2015	Huge quantity of Gutka seized	Jammu and Kashmir	https://www.greaterkashmir.com/news/kashmir/huge-quantity-of-gutka-seized/
Files\\213	06-07-2013	Officials destroy banned gutka products in Srinagar	Jammu and Kashmir	https://www.tribuneindia.com/2013/20130706/kashmir.htm#4
Files\\214	24-07-2018	Gutka worth Rs 35 lakh seized, two held	Maharashtra	https://www.business-standard.com/article/pti-stories/gutka-worth-rs-35-lakh-seized-two-held-118072400489_1.html
Files\\216	30-03-2013	Shimla police seize 12,000 gutka packets	Himachal Pradesh	https://sunpost.in/shimla-police-seize-12000-gutka-packets/
Files\\219	04-05-2013	Police seize 2,000 kg of banned gutka, two arrested	New Delhi	https://www.ndtv.com/delhi-news/police-seize-2-000-kg-of-banned-gutka-two-arrested-521182
Files\\220	10-08-2019	Illegal gutka, pan masala units unearthed in Odisha; one held	Odisha	https://www.thehindu.com/news/cities/kolkata/illegal-gutka-pan-masala-units-unearthed-in-odisha-one-held/article28970789.ece
Files\\221	07-09-2014	Cops Raid Gutka Units, Seize Rs 1 Cr Material	Odisha	https://www.newindianexpress.com/states/odisha/2014/sep/07/Cops-Raid-Gutka-Units-Seize-Rs-1-Cr-Material-657463.html
