# Supplementary material for: How does Indian news media report smokeless tobacco control? A content analysis of the gutka ban enforcement
Source: PLOS Glob Public Health. 2023 Mar 27;3(3):e0001724. doi: 10.1371/journal.pgph.0001724 (PMC10042338; doi:10.1371/journal.pgph.0001724)
Supplement: S2 Table — (PDF) [file pgph.0001724.s002.pdf]

S2 Table. Characteristics of the included news media reports.

Media files	Name of publication	News type	News content	Language	By-line	Slant	Beat	Administrative focus	Picture	Enforcement timing
Files\\001	The Times of India	Newspaper	News Article	English	Bureau	Positive	Crime	State	Representational: law enforcement	Transporting
Files\\002	The Hans India	Newspaper	News Article	English	Bureau	Positive	Crime	District/City	Seized gutka products	Storing
Files\\003	The Hindu	Newspaper	News Article	English	Staff Reporter	Positive	Crime	District/City	No picture	Manufacturing
Files\\004	The New Indian Express	Newspaper	News Article	English	Bureau	Positive	Crime	District/City	Representational: consuming gutka	Transporting
Files\\005	The Hans India	Newspaper	News Article	English	Roja Mayabrahma	Positive	Crime	District/City	Gutka products on display	Selling
Files\\006	Deccan Chronicle	Newspaper	News Article	English	Bureau	Neutral	City	State	Pan shop	Not Applicable
Files\\007	United News of India	News Agency	News Article	English	Bureau	Positive	Crime	District/City	No picture	Transporting
Files\\008	The Hans India	Newspaper	News Article	English	Bureau	Positive	Crime	District/City	Gutka manufacturing unit	Manufacturing
Files\\009	Deccan Chronicle	Newspaper	News Article	English	Bureau	Positive	Crime	District/City	Enforcement team with seized gutka products	Storing
Files\\010	The New Indian Express	Newspaper	News Article	English	Bureau	Positive	Crime	District/City	Seized ganja products	Storing
Files\\011	The Hans India	Newspaper	News Article	English	Srinivas Rao	Positive	Crime	State	Enforcement team with accused and seized gutka products	Transporting
Files\\012	The New Indian Express	Newspaper	News Article	English	Bureau	Positive	Crime	State	No picture	Transporting
Files\\013	The Hans India	Newspaper	News Article	English	Bureau	Positive	Crime	District/City	Enforcement team with accused and seized gutka products	Unassigned
Files\\014	The Hans India	Newspaper	News Article	English	Bureau	Positive	Crime	State	Enforcement team with seized gutka products	Manufacturing
Files\\015	The New Indian Express	Newspaper	News Article	English	Bureau	Positive	Crime	District/City	Enforcement activity	Selling
Files\\016	The Hans India	Newspaper	News Analysis	English	Bureau	Positive	Crime	State	Seized gutka products	Not Applicable
Files\\017	The Hans India	Newspaper	News Article	English	Bureau	Neutral	Crime	State	Representational: consuming gutka	Transporting
Files\\018	The Hans India	Newspaper	News Article	English	Bureau	Positive	Crime	District/City	Enforcement team with seized gutka products	Storing

Files\\019	The Hindu	Newspaper	News Article	English	Special Correspondent	Positive	Crime	State	Enforcement team with seized gutka products	Storing
Files\\020	The Hans India	Newspaper	News Article	English	Bureau	Positive	Crime	State	Representational: handcuffs	Unassigned
Files\\021	The Hans India	Newspaper	News Analysis	English	P V Prasad	Positive	Crime	State	Gutka products on display	Transporting and Storing
Files\\022	The Hans India	Newspaper	News Analysis	English	K Praveen Kumar	Positive	Crime	State	Seized gutka products	Transporting
Files\\023	The Telegraph	Newspaper	News Article	English	Bureau	Mixed	City	District/City	No picture	Unassigned
Files\\024	The Sentinel	Newspaper	News Analysis	English	Bureau	Positive	City	District/City	No picture	Transporting and Selling
Files\\026	The Times of India	Newspaper	News Article	English	Bureau	Positive	City	State	Representational: consuming gutka	Selling
Files\\027	NDTV	Newsmedia Company	News Article	English	PTI	Positive	City	State	Representational: jail	Unassigned
Files\\028	Hindustan Dainik	Newspaper	News Article	Hindi	Bureau	Positive	Crime	District/City	No picture	Transporting
Files\\029	Hindustan Dainik	Newspaper	News Article	Hindi	Bureau	Positive	Crime	District/City	No picture	Transporting
Files\\030	Dainik Jagran	Newspaper	News Article	Hindi	Bureau	Positive	Crime	District/City	Enforcement team with seized gutka products	Transporting
Files\\031	Patna Live	News Portal	News Article	Hindi	Bureau	Positive	City	State	No picture	Transporting
Files\\032	The New Indian Express	Newspaper	News Analysis	English	Bureau	Positive	Crime	State	Representational: handcuffs	Storing
Files\\033	Naidunia	News Portal	News Article	Hindi	Bureau	Positive	Crime	District/City	Enforcement team with seized gutka products	Storing
Files\\034	Naidunia	News Portal	News Article	Hindi	Bureau	Mixed	Crime	District/City	Gutka products on display	Transporting and Selling
Files\\036	Naidunia	News Portal	News Article	Hindi	Bureau	Positive	Crime	District/City	Seized gutka products	Selling and Storing
Files\\037	Dainik Bhaskar	Newspaper	News Article	Hindi	Bureau	Positive	Crime	District/City	No picture	Transporting and Storing
Files\\038	Hari Bhoomi	Newspaper	News Article	Hindi	Bureau	Positive	City	District/City	No picture	Selling
Files\\039	Patrika	Newspaper	News Article	Hindi	Kanchan Jwala	Positive	Crime	State	Enforcement team with seized gutka products	Storing
Files\\040	Patrika	Newspaper	News Article	Hindi	Kanchan Jwala	Positive	Crime	State	Pan shop	Storing
Files\\041	Dainik Bhaskar	Newspaper	News Article	Hindi	Bureau	Positive	Crime	State	No picture	Storing
Files\\042	Naidunia	News Portal	News Article	Hindi	Bureau	Positive	City	District/City	No picture	Storing
Files\\044	The Voices	News Portal	News Article	Hindi	Ramesh Gupta	Positive	Crime	District/City	Enforcement team with seized gutka products	Storing

Files\\045	Glibs	News Portal	News Article	Hindi	Bureau	Positive	Crime	District/City	Enforcement team with seized gutka products	Selling
Files\\046	Daijiworld	News Portal	News Article	English	Bureau	Positive	City	State	No picture	Selling and Storing
Files\\047	The Times of India	Newspaper	News Article	English	Bureau	Positive	City	State	No picture	Selling and Storing
Files\\048	The Times of India	Newspaper	News Article	English	Bureau	Positive	Crime	State	No picture	Transporting
Files\\049	The Navhind Times	Newspaper	News Analysis	English	Bureau	Neutral	Crime	State	No picture	Selling
Files\\052	The Goan Everyday	Newspaper	News Analysis	English	Bureau	Positive	City	State	No picture	Selling
Files\\054	The Times of India	Newspaper	News Article	English	Bureau	Positive	City	District/City	Gutka manufacturing unit	Manufacturing
Files\\055	The Indian Express	Newspaper	News Analysis	English	Bureau	Positive	Crime	State	Gutka products on display	Selling
Files\\059	The Times of India	Newspaper	News Analysis	English	Bureau	Positive	Crime	State	Representational: consuming gutka	Manufacturing
Files\\060	DeshGujarat	News Portal	News Article	English	Bureau	Positive	Crime	District/City	No picture	Transporting
Files\\063	The Times of India	Newspaper	News Analysis	English	Bureau	Positive	Crime	State	No picture	Selling and Storing
Files\\064	Hindustan Times	Newspaper	News Article	English	HT Correspondent	Positive	City	State	No picture	Selling
Files\\065	The Tribune	Newspaper	News Article	English	B S Malik	Positive	Crime	District/City	No picture	Manufacturing
Files\\067	Himachal Watcher	News Portal	News Analysis	English	Bureau	Neutral	City	State	Enforcement activity	Selling
Files\\068	The Economic Times	Newspaper	News Article	English	PTI	Positive	Crime	State	Representational: consuming gutka	Manufacturing
Files\\069	Patrika	Newspaper	News Article	Hindi	Nitin Sadaphal	Positive	Crime	District/City	Enforcement team with accused	Unassigned
Files\\070	News18	Newsmedia Company	News Article	Hindi	Manoj Khandekar	Positive	Crime	State	Seized gutka products	Transporting
Files\\071	Lokmat	Newspaper	News Article	Marathi	Bureau	Positive	Crime	District/City	Enforcement team with accused and seized gutka products	Storing
Files\\072	Business Standard	Newspaper	News Analysis	English	PTI	Positive	Crime	District/City	No picture	Transporting and Storing
Files\\073	The Asian Age	Newspaper	News Article	English	Bureau	Positive	Crime	District/City	Enforcement team with seized gutka products	Transporting
Files\\074	Business Standard	Newspaper	News Analysis	English	PTI	Positive	Crime	State	No picture	Transporting

Files\\075	Sakal	Newspaper	News Article	Marathi	Dinesh Gogi	Positive	Crime	District/City	Enforcement team with seized gutka products	Unassigned
Files\\076	Down To Earth	News Magazine	News Analysis	English	Jyotsna Singh	Positive	Crime	State	Pan shop	Selling
Files\\077	The New Indian Express	Newspaper	News Article	English	PTI	Positive	Crime	District/City	Representational: handcuffs	Transporting
Files\\078	Maharashtra Times	Newspaper	News Article	Marathi	Bureau	Positive	Crime	State	Representational: consuming gutka	Transporting
Files\\079	The Indian Express	Newspaper	News Article	English	Bureau	Positive	Crime	District/City	Google map	Storing
Files\\082	The Times of India	Newspaper	News Analysis	English	Sumitra Debroy	Positive	Court	State	Representational: consuming gutka	Transporting
Files\\083	The Times of India	Newspaper	News Article	English	Bureau	Positive	Crime	District/City	Representational: law enforcement	Storing
Files\\084	The Times of India	Newspaper	News Analysis	English	Bureau	Positive	Crime	District/City	Representational: other tobacco products	Storing
Files\\085	The Times of India	Newspaper	News Analysis	English	PTI	Positive	Crime	District/City	Representational: handcuffs	Transporting
Files\\086	The Times of India	Newspaper	News Analysis	English	Bureau	Positive	Crime	State	Representational: handcuffs	Transporting
Files\\087	The Times of India	Newspaper	News Analysis	English	Umesh Ishalkar	Positive	Crime	District/City	No picture	Selling
Files\\089	Hindustan Times	Newspaper	News Analysis	English	HT Correspondent	Positive	Crime	State	Pan shop	Not Applicable
Files\\091	The Times of India	Newspaper	News Analysis	English	Bureau	Positive	Crime	District/City	Representational: handcuffs	Storing
Files\\092	Lokmat	Newspaper	News Article	Marathi	Bureau	Positive	Crime	District/City	Accused and seized gutka products	Transporting
Files\\094	The Indian Express	Newspaper	News Analysis	English	PTI	Positive	Crime	State	Enforcement activity	Not Applicable
Files\\095	Sakal	Newspaper	News Article	Marathi	Bureau	Positive	Crime	State	Enforcement team with seized gutka products	Transporting
Files\\096	Lokmat	Newspaper	News Article	Marathi	Bureau	Positive	Crime	District/City	Seized gutka products	Transporting
Files\\098	The Times of India	Newspaper	News Article	English	Bureau	Positive	Crime	District/City	Representational: consuming gutka	Selling
Files\\099	The Indian Express	Newspaper	News Article	English	PTI	Positive	Crime	State	Enforcement activity	Transporting
Files\\100	MoneyLife	Financial Magazine	News Analysis	English	PTI	Positive	Crime	District/City	No picture	Not Applicable
Files\\101	The Times of India	Newspaper	News Article	English	Bureau	Positive	Crime	District/City	Representational: law enforcement	Storing

Files\\102	Lokmat	Newspaper	News Article	Marathi	Bureau	Positive	Crime	State	Representational: city	Transporting
Files\\103	Business Standard	Newspaper	News Article	English	PTI	Positive	Crime	State	No picture	Transporting
Files\\104	United News of India	News Agency	News Article	English	Bureau	Positive	Crime	District/City	No picture	Storing
Files\\105	Sambad	Newspaper	News Article	English	Bureau	Positive	Crime	District/City	Seized gutka products	Transporting
Files\\106	Odisha Bytes	News Portal	News Article	English	Bureau	Positive	Crime	District/City	Gutka products on display	Transporting
Files\\108	Eastern Mirror	Newspaper	News Article	English	PTI	Positive	City	State	No picture	Unassigned
Files\\109	Khas Khabar	News Portal	News Article	Hindi	Bureau	Positive	Crime	District/City	Representational: consuming gutka	Manufacturing
Files\\110	The Hindu	Newspaper	News Article	English	PTI	Positive	Crime	State	Gutka products on display	Not Applicable
Files\\111	The Hindu	Newspaper	News Article	English	Staff Reporter	Positive	Crime	State	Enforcement team with seized gutka products	Transporting
Files\\112	The Times of India	Newspaper	News Article	English	Siddarth Prabhakar	Neutral	Crime	State	Enforcement team with seized gutka products	Transporting
Files\\113	The Hindu	Newspaper	News Article	English	D Madhavan	Positive	Crime	State	Enforcement team with seized gutka products	Transporting
Files\\114	India Today	News Magazine	News Article	English	PTI	Positive	Crime	District/City	No picture	Selling
Files\\115	The Times of India	Newspaper	News Article	English	Bureau	Positive	Crime	District/City	No picture	Storing
Files\\116	The Times of India	Newspaper	News Article	English	Bureau	Positive	Crime	District/City	Enforcement team with accused and seized gutka products	Transporting
Files\\117	The Hindu	Newspaper	News Article	English	Special Correspondent	Positive	Crime	District/City	Seized gutka products	Transporting
Files\\118	Dina Thanthi	Newspaper	News Article	English	Bureau	Positive	Crime	District/City	Gutka products on display	Transporting and Storing
Files\\119	News Today	Newspaper	News Article	English	Bureau	Positive	Crime	District/City	Representational: consuming gutka	Storing
Files\\121	The Hindu	Newspaper	News Article	English	Staff Reporter	Positive	Crime	District/City	No picture	Storing
Files\\122	The New Indian Express	Newspaper	News Article	English	R Shivakumar	Positive	Crime	District/City	Representational: handcuffs	Transporting
Files\\123	The Times of India	Newspaper	News Article	English	Bureau	Positive	Crime	District/City	No picture	Storing
Files\\124	The Times of India	Newspaper	News Article	English	Bureau	Positive	Crime	District/City	Representational: consuming gutka	Storing
Files\\125	The Times of India	Newspaper	News Analysis	English	Bureau	Positive	City	State	No picture	Not Applicable
Files\\126	The Times of India	Newspaper	News Article	English	Deepak Karthik	Positive	City and Crime	District/City	Representational: law enforcement	Transporting

Files\\127	The Times of India	Newspaper	News Article	English	Bureau	Positive	Crime	District/City	Enforcement team with seized gutka products	Transporting
Files\\128	The Times of India	Newspaper	News Article	English	Nivedha Selvam	Positive	Crime	District/City	Representational: consuming gutka	Storing
Files\\129	Deccan Chronicle	Newspaper	News Article	English	Bureau	Positive	Crime	District/City	Enforcement activity	Storing
Files\\130	The Times of India	Newspaper	News Article	English	Bureau	Positive	Crime	District/City	Representational: law enforcement	Storing
Files\\131	The Times of India	Newspaper	News Article	English	Balajee C R	Positive	City	District/City	Enforcement team with seized gutka products	Transporting and Selling and Storing
Files\\132	The Times of India	Newspaper	News Analysis	English	Bureau	Positive	City and Crime	District/City	Enforcement team with seized gutka products	Storing
Files\\133	Deccan Chronicle	Newspaper	News Article	English	Bureau	Positive	Crime	District/City	Gutka products on display	Transporting
Files\\134	Deccan Chronicle	Newspaper	News Article	English	Bureau	Positive	Crime	District/City	Representational: handcuffs	Transporting
Files\\135	The Times of India	Newspaper	News Article	English	Bureau	Positive	Crime	District/City	Representational: handcuffs	Storing
Files\\136	The Times of India	Newspaper	News Article	English	Bureau	Positive	Crime	District/City	Representational: consuming gutka	Storing
Files\\137	The Hindu	Newspaper	News Analysis	English	Special Correspondent	Positive	Health	District/City	No picture	Transporting
Files\\138	The Times of India	Newspaper	News Article	English	Balajee C R	Positive	Crime	District/City	Representational: law enforcement	Storing
Files\\139	The New Indian Express	Newspaper	News Article	English	Bureau	Neutral	Crime	District/City	Representational: consuming gutka	Not Applicable
Files\\140	News Today	Newspaper	News Article	English	Bureau	Positive	Crime	District/City	Seized gutka products	Transporting
Files\\141	The Times of India	Newspaper	News Analysis	English	Siddarth Prabhakar and Ekatha Ann John	Positive	Crime	District/City	Enforcement team with seized gutka products	Not Applicable
Files\\142	The Times of India	Newspaper	News Article	English	Bureau	Positive	Crime	District/City	Representational: consuming gutka	Transporting and Storing
Files\\143	The Times of India	Newspaper	News Analysis	English	Bureau	Positive	Crime	District/City	Pan shop	Transporting
Files\\144	The Times of India	Newspaper	News Analysis	English	A Subburaj	Positive	Crime	District/City	Representational: consuming gutka	Manufacturing and Storing
Files\\145	Deccan Chronicle	Newspaper	News Article	English	Bureau	Positive	Crime	District/City	Gutka products on display	Selling
Files\\146	The Times of India	Newspaper	News Article	English	Bureau	Positive	City	District/City	No picture	Storing

Files\\147	The Times of India	Newspaper	News Article	English	Bureau	Positive	City and Crime	District/City	Representational: warning sign	Selling
Files\\148	Mirror Now	Newsmedia Company	News Analysis	English	Bureau	Positive	Crime	State	No picture	Transporting
Files\\149	The New Indian Express	Newspaper	News Analysis	English	Sahaya Novinston Lobo	Mixed	Crime	District/City	Image of the victim	Selling
Files\\150	The Times of India	Newspaper	News Article	English	Siddarth Prabhakar	Positive	Crime	District/City	No picture	Transporting
Files\\151	Deccan Chronicle	Newspaper	News Article	English	Bureau	Positive	Crime	District/City	Enforcement team with seized gutka products	Transporting
Files\\152	The Times of India	Newspaper	News Analysis	English	Ekatha Ann John	Neutral	City and Crime	State	Gutka products on display	Not Applicable
Files\\153	Deccan Chronicle	Newspaper	News Article	English	Bureau	Positive	Crime	District/City	Pan shop	Selling
Files\\154	The Covai Post	News Portal	News Article	English	Bureau	Positive	City	District/City	Enforcement team with seized gutka products	Storing
Files\\155	Deccan Chronicle	Newspaper	News Article	English	Bureau	Positive	Crime	District/City	Representational: Ganja	Selling
Files\\156	GNS News	News Agency	News Article	English	Lucky Jain	Neutral	City	District/City	Representational: warning sign	Storing
Files\\158	The Hindu	Newspaper	News Analysis	English	Staff Reporter	Positive	City	District/City	Seized gutka products	Storing
Files\\159	Deccan Chronicle	Newspaper	News Article	English	Bureau	Positive	Crime	District/City	Gutka products on display	Transporting
Files\\160	Telangana Today	Newspaper	News Article	English	Bureau	Positive	Crime	District/City	No picture	Selling
Files\\163	The Hans India	Newspaper	News Article	English	Bureau	Positive	Crime	District/City	Enforcement team with seized gutka products	Storing
Files\\164	The Hans India	Newspaper	News Article	English	Bureau	Positive	Crime	District/City	Enforcement team with seized gutka products	Transporting
Files\\165	The Hans India	Newspaper	News Article	English	Bureau	Positive	Crime	District/City	Enforcement team with seized gutka products	Transporting
Files\\166	The Hindu	Newspaper	News Article	English	Staff Reporter	Positive	Crime	District/City	Enforcement team with seized gutka products	Transporting
Files\\167	United News of India	News Agency	News Article	English	Bureau	Positive	Crime	District/City	No picture	Transporting
Files\\168	The New Indian Express	Newspaper	News Article	English	Bureau	Neutral	Crime	District/City	Representational: other tobacco products	Transporting
Files\\169	Telangana Today	Newspaper	News Article	English	Bureau	Positive	Crime	District/City	No picture	Transporting
Files\\170	Deccan Chronicle	Newspaper	News Article	English	Bureau	Positive	Crime	District/City	Gutka products on display	Transporting and Storing

Files\\171	The Hans India	Newspaper	News Article	English	Bureau	Positive	Crime	District/City	Accused	Storing
Files\\172	The Times of India	Newspaper	News Analysis	English	PTI	Neutral	Crime	District/City	Representational: handcuffs	Storing
Files\\173	Telangana Today	Newspaper	News Article	English	Bureau	Neutral	Crime	District/City	Enforcement team with seized gutka products	Transporting
Files\\174	The Hans India	Newspaper	News Article	English	Yanadi Reddy Mattipati	Neutral	Crime	District/City	Seized gutka products	Storing
Files\\175	The Hans India	Newspaper	News Article	English	Bureau	Positive	Crime	District/City	Representational: warning sign	Selling
Files\\176	Telangana Today	Newspaper	News Article	English	Bureau	Positive	Crime	District/City	No picture	Storing
Files\\177	The Hans India	Newspaper	News Analysis	English	Bureau	Positive	Health	State	Gutka products on display	Not Applicable
Files\\179	The Hans India	Newspaper	News Article	English	Yanadi Reddy Mattipati	Positive	Crime	District/City	Enforcement team with seized gutka products	Selling
Files\\180	The Hans India	Newspaper	News Article	English	Bureau	Positive	Crime	District/City	Enforcement team with accused and seized gutka products	Transporting
Files\\182	The Hans India	Newspaper	News Article	English	Bureau	Positive	Crime	District/City	Enforcement activity	Selling
Files\\183	The Hans India	Newspaper	News Article	English	Bureau	Positive	Crime	District/City	Seized gutka products	Selling
Files\\184	Deccan Chronicle	Newspaper	News Article	English	Bureau	Mixed	City and Crime	State	Seized money	Transporting
Files\\185	Telangana Today	Newspaper	News Article	English	Bureau	Positive	Crime	District/City	Enforcement team with accused and seized gutka products	Transporting
Files\\186	The Hindu	Newspaper	News Article	English	Staff Reporter	Positive	Crime	State	Seized gutka products	Transporting
Files\\187	The Hans India	Newspaper	News Article	English	Bureau	Positive	Crime	District/City	Enforcement team with accused and seized gutka products	Transporting
Files\\188	Sakshi Samachar	News Portal	News Article	Hindi	Bureau	Positive	Crime	District/City	Enforcement team with accused	Transporting
Files\\189	Deccan Chronicle	Newspaper	News Article	English	Bureau	Neutral	Crime	District/City	Representational: handcuffs	Selling
Files\\190	Deccan Chronicle	Newspaper	News Article	English	Bureau	Neutral	Crime	District/City	Representational: handcuffs	Transporting
Files\\191	The Hans India	Newspaper	News Article	English	Bureau	Positive	Crime	District/City	Enforcement team with accused and seized gutka products	Selling

Files\\192	Telangana Today	Newspaper	News Article	English	Bureau	Positive	Crime	District/City	Enforcement team with accused and seized gutka products	Transporting and Storing
Files\\193	Telangana Today	Newspaper	News Article	English	Bureau	Positive	Crime	District/City	Enforcement team with seized gutka products	Transporting and Storing
Files\\194	The Hindu	Newspaper	News Article	English	M Rajeev	Mixed	City and Crime	State	Enforcement activity	Unassigned
Files\\195	The Times of India	Newspaper	News Article	English	Bureau	Positive	Crime	District/City	No picture	Transporting and Storing
Files\\197	The Northeast Today	News Portal	News Article	English	Bureau	Positive	Crime	District/City	Gutka products on display	Transporting
Files\\198	The Times of India	Newspaper	News Article	English	Bureau	Positive	Crime	District/City	No picture	Storing
Files\\199	Business Standard	Newspaper	News Analysis	English	PTI	Mixed	City	District/City	No picture	Unassigned
Files\\200	The Times of India	Newspaper	News Article	English	Bureau	Positive	Crime	District/City	Representational: handcuffs	Storing
Files\\201	The Times of India	Newspaper	News Article	English	Santosh Sonawane	Positive	Crime	District/City	No picture	Transporting
Files\\202	The Times of India	Newspaper	News Article	English	Bureau	Positive	Crime	District/City	No picture	Transporting
Files\\203	The Times of India	Newspaper	News Analysis	English	Bureau	Positive	City	District/City	Gutka products on display	Transporting
Files\\204	The Times of India	Newspaper	News Article	English	Bureau	Positive	City	District/City	No picture	Storing
Files\\205	The Times of India	Newspaper	News Article	English	Bureau	Positive	Crime	District/City	No picture	Storing
Files\\206	The New Indian Express	Newspaper	News Article	English	Aravind Raj	Positive	Crime	District/City	No picture	Storing
Files\\207	The New Indian Express	Newspaper	News Article	English	Bureau	Positive	Crime	District/City	Representational: consuming gutka	Manufacturing
Files\\208	The Times of India	Newspaper	News Article	English	Bureau	Positive	Crime	District/City	No picture	Unassigned
Files\\209	The Hans India	Newspaper	News Article	English	Bureau	Positive	Crime	District/City	Seized gutka products	Selling
Files\\210	The Hans India	Newspaper	News Article	English	Bureau	Positive	Crime	District/City	Seized gutka products	Selling
Files\\211	Telangana Today	Newspaper	News Article	English	Bureau	Positive	City	District/City	Enforcement team with accused and seized gutka products	Selling
Files\\212	Greater Kashmir	Newspaper	News Article	English	Bureau	Positive	City	District/City	No picture	Selling
Files\\213	The Tribune	Newspaper	News Article	English	Bureau	Positive	City	District/City	Enforcement activity	Selling
Files\\214	Business Standard	Newspaper	News Article	English	PTI	Positive	Crime	District/City	No picture	Transporting
Files\\216	Sun Post	News Portal	News Analysis	English	Baladev S Chauhan	Neutral	Crime	District/City	Gutka products on display	Transporting

Files\\219	NDTV	Newsmedia Company	News Analysis	English	PTI	Positive	Crime	District/City	No picture	Storing
Files\\220	The Hindu	Newspaper	News Article	English	Staff Reporter	Positive	Crime	State	No picture	Manufacturing and Transport
Files\\221	The New Indian Express	Newspaper	News Analysis	English	Bureau	Positive	Crime	District/City	No picture	Manufacturing
