# Supplementary material for: How does Indian news media report smokeless tobacco control? A content analysis of the gutka ban enforcement
Source: PLOS Glob Public Health. 2023 Mar 27;3(3):e0001724. doi: 10.1371/journal.pgph.0001724 (PMC10042338; doi:10.1371/journal.pgph.0001724)
Supplement: S3 Table — (PDF) [file pgph.0001724.s003.pdf]

S3 Table. State-wise quantity and amount worth of gutka seized.

Media file	Location	Quantity seized	Amount worth
Files\\002	Andhra Pradesh		6.22 Lakh
Files\\004	Andhra Pradesh	250 bags	10 Lakh
Files\\007	Andhra Pradesh	45 bags	10 Lakh
Files\\008	Andhra Pradesh	6 machines and raw materials, 22 bags, 58 bags	25 Lakh
Files\\009	Andhra Pradesh		6 Lakh
Files\\010	Andhra Pradesh		16 Lakh
Files\\011	Andhra Pradesh		5.50 Lakh
Files\\012	Andhra Pradesh	68,000 packets	10 Lakh
Files\\013	Andhra Pradesh	88,674 packets, 2,150 gms	15 Lakh
Files\\014	Andhra Pradesh	40 packets cigarette, machines	4 Lakh
Files\\015	Andhra Pradesh		1 Lakh
Files\\016	Andhra Pradesh		30 Lakh
Files\\017	Andhra Pradesh	23 bags	23 Lakh
Files\\018	Andhra Pradesh		4 Lakh
Files\\019	Andhra Pradesh	265 gunny bags of tobacco powder	3 crores
Files\\021	Andhra Pradesh		22-25 Lakh
Files\\177	Andhra Pradesh		
Files\\195	Andhra Pradesh	150 bags	7-21 Lakh
Files\\197	Arunachal Pradesh		
Files\\023	Assam		
Files\\024	Assam	4 bags, 20 bags, 80 bags	
Files\\027	Bihar	20 gms	
Files\\028	Bihar	200 bags	7 Lakh
Files\\029	Bihar	150 carton, 75 carton	
Files\\030	Bihar		40 Lakh
Files\\031	Bihar		22 Lakh
Files\\064	Chandigarh	5000 pouches	
Files\\032	Chhattisgarh		50 Lakh
Files\\033	Chhattisgarh	12 bags	6 Lakh
Files\\034	Chhattisgarh	6 bags, 26 bags, 24 bags	1.5 crores
Files\\036	Chhattisgarh	30 bags	10 Lakh
Files\\037	Chhattisgarh	95 bags, 8 bags	53.5 Lakh
Files\\038	Chhattisgarh	266 bags	27 thousand
Files\\039	Chhattisgarh	46 bags	10 Lakh
Files\\040	Chhattisgarh	100 bags, 46 bags, 20 thousand packets	50 Lakh
Files\\041	Chhattisgarh	15 bags, 9 bags (900 packets), 13 bags (1300 packets)	10 Lakh
Files\\042	Chhattisgarh	21 bags	34 Lakh
Files\\044	Chhattisgarh		
Files\\045	Chhattisgarh	12 cartons	15 Lakh
Files\\046	Goa		9.84 Lakh
Files\\047	Goa		9.85 Lakh, 1.2 Lakh
Files\\048	Goa		3 Lakh
Files\\049	Goa	200 bags, 1.5 kg	
Files\\052	Goa		2,000
Files\\054	Gujarat		70 crores

Files\\055	Gujarat		
Files\\059	Gujarat		8 crores
Files\\060	Gujarat		
Files\\063	Gujarat	67,500 sachets	2.5 Lakh
Files\\079	Gujarat	65 carton (12,000 packets)	15.60 Lakh
Files\\065	Haryana	7.50 quintals	
Files\\067	Himachal Pradesh	9 sacks (150 kg)	
Files\\216	Himachal Pradesh	12,000 packets	
Files\\212	Jammu and Kashmir		15,000
Files\\213	Jammu and Kashmir		15,000
Files\\068	Karnataka		
Files\\069	Madhya Pradesh	59 bags, 3 bags	15 Lakh, 54000
Files\\070	Madhya Pradesh		75 Lakh
Files\\026	Maharashtra		1 Lakh
Files\\071	Maharashtra	8271 packets, 85 bags	10,27,640
Files\\072	Maharashtra		51.83 Lakh
Files\\073	Maharashtra		9.98 Lakh
Files\\074	Maharashtra		1.77 crores
Files\\075	Maharashtra	15 bags	6.5 Lakh
Files\\076	Maharashtra		32 crores
Files\\077	Maharashtra		44 Lakh
Files\\078	Maharashtra		1.5 crores, 39,54,600, 10,80,000
Files\\082	Maharashtra		203 crores
Files\\083	Maharashtra		28 Lakh
Files\\084	Maharashtra		8.50 Lakh
Files\\085	Maharashtra		1 crores
Files\\086	Maharashtra		12 Lakh
Files\\087	Maharashtra		7.41 crores
Files\\089	Maharashtra		171 crores, 20 crores
Files\\091	Maharashtra		30 Lakh, 74 Lakh
Files\\092	Maharashtra	30 bags, 35 bags, 1 bag	9 Lakh, 10,40,000, 31,000
Files\\094	Maharashtra		114.2 crores
Files\\095	Maharashtra	150 bags, 290 bags	2 crores
Files\\096	Maharashtra	100 bags	32 Lakh, 12 Lakh
Files\\098	Maharashtra	2.44 kg	1,665
Files\\099	Maharashtra		12.28 Lakh
Files\\100	Maharashtra	5 tonnes	
Files\\101	Maharashtra		4.52 Lakh
Files\\102	Maharashtra		1 crore
Files\\103	Maharashtra		70 Lakh
Files\\104	Maharashtra		11.56 Lakh
Files\\198	Maharashtra		25 Lakh
Files\\199	Maharashtra		1.4 crores
Files\\200	Maharashtra		34 Lakh, 19.5 Lakh
Files\\201	Maharashtra		6 Lakh

Files\\202	Maharashtra		22 Lakh
Files\\203	Maharashtra	243 bags	38 Lakh
Files\\214	Maharashtra	153 gunny bags	35 Lakh
Files\\108	Nagaland		16.72 Lakh
Files\\219	New Delhi	2,000 kg	
Files\\105	Odisha	30 sacks	
Files\\106	Odisha	50 sacks	
Files\\220	Odisha	48 bags seized, 50 sacks	
Files\\221	Odisha	86 sacks (5.16 Lakh sachets)	15 Lakh.
Files\\109	Rajasthan		70 Lakh
Files\\110	Rajasthan		
Files\\111	Tamil Nadu	6 tonnes, 160 bags each of 40 kg, 16 tones	
Files\\112	Tamil Nadu	287kg, 200 bundles, 12 sacks, 2,400 kg	6 Lakh, 15 Lakh
Files\\113	Tamil Nadu	3 tonnes, 100 bags 30 kg each, 16 tonnes	
Files\\114	Tamil Nadu	750 kg	
Files\\115	Tamil Nadu	300 kg	3 Lakh
Files\\116	Tamil Nadu	840 kg, 648 kg	6.44 Lakh
Files\\117	Tamil Nadu	150 bags	22.5 Lakh
Files\\118	Tamil Nadu		4 Lakh
Files\\119	Tamil Nadu	6 tonnes	5.37 Lakh
Files\\121	Tamil Nadu	2 tonnes	4 Lakh
Files\\122	Tamil Nadu	54 bundles	50 Lakh
Files\\123	Tamil Nadu	2 tonnes	3 Lakh
Files\\124	Tamil Nadu	575 kg	5 Lakh
Files\\125	Tamil Nadu		
Files\\126	Tamil Nadu	2,500 kg, 90 boxes	
Files\\127	Tamil Nadu	50 bags, 30 kg	7.5 Lakh
Files\\128	Tamil Nadu	2,350 kg	13.72 Lakh
Files\\129	Tamil Nadu	5 tonnes	
Files\\130	Tamil Nadu	6.5 tonnes	
Files\\131	Tamil Nadu	250 kg, 50 kg, 200 kg	2 Lakh
Files\\132	Tamil Nadu	1.5 tonnes, 3 tonnes	10 Lakh
Files\\133	Tamil Nadu	1.5 tonnes	
Files\\134	Tamil Nadu		11 Lakh, 17 Lakh
Files\\135	Tamil Nadu	5 tonnes, 3.32 tonnes, 1,461 sachets	35 Lakh
Files\\136	Tamil Nadu	200 kg	2 Lakh
Files\\137	Tamil Nadu	120 kg	
Files\\138	Tamil Nadu	3600 kg	11 Lakh
Files\\139	Tamil Nadu	50 kg	75,000
Files\\140	Tamil Nadu	50 kg, 1360 kg	15 Lakh
Files\\141	Tamil Nadu	200 cartons	15 Lakh
Files\\142	Tamil Nadu	18 bundles	2.5 Lakh, 1 Lakh
Files\\143	Tamil Nadu	200 cartons	15 Lakh
Files\\144	Tamil Nadu	648 kg, 750 kg	3.24 Lakh
Files\\145	Tamil Nadu	6 kg, 400 packets	5000, 45,000
Files\\146	Tamil Nadu	210 kg	3 Lakh
Files\\147	Tamil Nadu	75 kg, 150 kg	1 Lakh, 2 Lakh
Files\\148	Tamil Nadu	5 tonnes in 70 bags	
Files\\149	Tamil Nadu	100 kg	4 Lakh

Files\\150	Tamil Nadu	240 kg	
Files\\151	Tamil Nadu		50 Lakh
Files\\152	Tamil Nadu		
Files\\153	Tamil Nadu	112 kg, 16 kg	5,000
Files\\154	Tamil Nadu	212 kg	2 Lakh
Files\\155	Tamil Nadu	3 kg, 1.2 kg	
Files\\156	Tamil Nadu	210 kg	3 Lakh
Files\\158	Tamil Nadu	1,000 kg	
Files\\159	Tamil Nadu	850 kg in 28 bundles	1 crore
Files\\204	Tamil Nadu		50000
Files\\205	Tamil Nadu	950 kg	
Files\\206	Tamil Nadu	1.5 tons in 30 bags, 15 tons	
Files\\207	Tamil Nadu	648 kg of gutka in 3 Lakh small packets, 79 kg, 1,398 kg	
Files\\208	Tamil Nadu	300 kg, 48.5 kg	
Files\\001	Telangana	230 gutka bags, 3.2 tons, each bag 70 packets	1.38 crores
Files\\003	Telangana	60,000 sachets	
Files\\005	Telangana		1 crore
Files\\006	Telangana		
Files\\020	Telangana	4 gunny bags; 13 bags of gutka	
Files\\022	Telangana	2 lorry loaded	30 Lakh
Files\\160	Telangana		7.9 Lakh
Files\\163	Telangana		15 Lakh
Files\\164	Telangana		21 Lakh, 21.85 Lakh, 27 Lakh
Files\\165	Telangana		1.5 Lakh
Files\\166	Telangana		15.12 Lakh, 1.43 crores
Files\\167	Telangana		1.5 Lakh
Files\\168	Telangana		1.43 crores
Files\\169	Telangana		5.63 Lakh
Files\\170	Telangana		13 Lakh, 16,000
Files\\171	Telangana		1.2 crores
Files\\172	Telangana		1.2 crores
Files\\173	Telangana		27 Lakh
Files\\174	Telangana		10.5 Lakh
Files\\175	Telangana		15 Lakh, 76,000
Files\\176	Telangana		1 crore
Files\\179	Telangana		3.25 Lakh
Files\\180	Telangana		70,000
Files\\182	Telangana	91 pouches, 109 pouches	
Files\\183	Telangana		10 Lakh
Files\\184	Telangana	29 kg	3.5 Lakh, 24 Lakh
Files\\185	Telangana	13 bags	26 Lakh, 26,000
Files\\186	Telangana	883 cartons	50 Lakh
Files\\187	Telangana		26 Lakh, 26,000
Files\\188	Telangana		1.5 crores
Files\\189	Telangana	1,457 packets, 1,287 sachets, 500gm	4,500
Files\\190	Telangana	18 bags	3 Lakh

Files\\191	Telangana	114 bags	57.35 Lakh, 40,000
Files\\192	Telangana		54.67 Lakh
Files\\193	Telangana	62 bags	12 Lakh
Files\\194	Telangana	2,038 kg, 1,080 kg	47.45 crores, 1.5 Lakh
Files\\209	Telangana		50 Lakh
Files\\210	Telangana	40 bags	
Files\\211	Telangana		3.25 Lakh
