# Supplementary material for: How does Indian news media report smokeless tobacco control? A content analysis of the gutka ban enforcement
Source: PLOS Glob Public Health. 2023 Mar 27;3(3):e0001724. doi: 10.1371/journal.pgph.0001724 (PMC10042338; doi:10.1371/journal.pgph.0001724)
Supplement: Attachment — Submitted filename: Response to Reviewers (Final).docx [file pgph.0001724.s004.docx]

Date: 10/01/2023

**Author Response Letter: PLOS Global Public Health [PGPH-D-22-00341]**

Dear Dr. Madhukar Pai, Editor-In-Chief, and the reviewers,

I am writing on behalf of all authors to submit the revised manuscript to PLOS Global Public Health. We appreciate the time and effort you and the reviewers put into providing feedback and making important improvements to our paper. We have prepared a point-by-point response to the comments and attached a marked and unmarked version of the manuscript accordingly.

In addition to peer-review, we rephrased the title of the manuscript to strongly reflect our study findings. The revised title reads as:

“What do Indian news media report during the enforcement of smokeless tobacco control? a content analysis of the gutka ban.”

**General Comments: (Overall quality of the paper)**

*In addition to addressing the comments from the two reviewers, please add a limitations section within the Discussion and explicitly discuss study limitations, including the methods used for media content analysis.*

**Response**: Thank you for this suggestion. We have included a section on ‘strengths and limitations’ within the discussion section starting Line no: 399.

**Journal requirements**

*Please send a completed ‘Competing Interests’ statement, including any COIs declared by your co-authors. If you have no competing interests to declare, please state “The authors have declared that no competing interests exist”.*

**Response**: We have updated the Competing Interests statement in the revised submission to: “The authors have declared that no competing interests exist.”

*Please amend your detailed Financial Disclosure statement. This is published with the article. It must therefore be completed in full sentences and contain the exact wording you wish to be published. A. State what role the funders took in the study. If the funders had no role in your study, please state: “The funders had no role in study design, data collection and analysis, decision to publish, or preparation of the manuscript.” B. If any authors received a salary from any of your funders, please state which authors and which funders. If you did not receive any funding for this study, please simply state: “The authors received no specific funding for this work.”*

**Response**: We have made the required changes in the revised submission as follows: “This work was supported by the DBT/Wellcome Trust India Alliance Early Career Fellowship [IA/CPHE/17/1/503338] awarded to Pragati B Hebbar. The funders had no role in study design, data collection and analysis, decision to publish, or preparation of the manuscript.”

*Guidelines for resubmitting your figure files are available below the reviewer comments at the end of this letter.*

**Response**: We have included figure files (1-5) based on the guidelines suggested by the journal.

**Reviewers’ comments**

**Reviewer 1:**

*Dear authors, my appreciations for taking up this vital study, with key findings. The study has been well drafted and the effort is commendable. The authors have done extensive literature work for the article and it is reflected in the quality of the manuscript. Except for a few minor revisions, the article is well-written.*

**Response**: Thank you for the positive feedback!

*Abstract: Although the objective states that the study aims to understand how the news shape public opinion, the results mentioned in the abstract does not reflect upon the impact on public opinion. I suggest the objective to be reworded.*

**Response**: Thank you for pointing this out. We have reworded the objectives in the Abstract and Introduction sections of the manuscript. The revised text reads as follows:

**Abstract**: “The purpose of this study was to look at how enforcement of gutka ban is covered in Indian news media and if media is a reliable source of data.” (Line nos: 29-30, Page no: 2)

**Introduction**: “In light of growing industry-media relations and increasing use of digital media services, how has Indian news media reported the enforcement of the gutka ban? As part of our ongoing study on the implementation of tobacco control policies in India [25], we aim to analyze patterns in the coverage of the ban and examine whether the media is a reliable source of enforcement-related data.” (Line nos: 80-84, Page no: 4)

*Results: The result section can be minimised to just stating the outcomes of the study rather than adding any descriptions. All the description and explanation are to be added to the discussion part. Pictorial or figure representation of the overall result might give an prompt idea of the major outcome of this study to the readers.*

**Response**: Thank you for the feedback. Our revised results section states the outcomes of the study and the discussion section explains the interpretations.

*Discussion: While there are ample results reported, the discussion feels inadequate. The authors can consider reducing the content on strengths and limitation, while discussion few more key components from results.*

**Response**: Thank you for your feedback. In the discussion section, we have added additional components based on the quantitative assessment of the news stories.

*Figures: The figures are nowhere cited in the main text, which makes it difficult to find the relevance of the figures to the manuscript.*

**Response**: Thank you for pointing this out. We have cited the figures in the main text. They are as follows:

Fig 1. Steps involved in identifying, screening, and assessing the eligibility of news reports. (Line no: 137, Page no:7)

Fig 2. Gutka seizures at manufacture units, storage facilities, and shops as reported in the media. (Line nos: 264-267, Page no: 15)

Fig 3. Illicit transport of gutka in Karnataka and Gujarat as reported in the media. (Line nos: 268-270, Page no: 15)

Fig 4. Gutka supply-chain linking people, processes, and infrastructure to end users in India. (Line nos: 288-289, Page no: 17)

Fig 5. How does Indian news media report gutka ban-enforcement? (Line no: 308, Page no: 18)

**Reviewer 2**

*Thank you for this important/useful analysis and novel approach in understanding the implementation of tobacco control laws. I have some minor suggestions to strengthen the paper and connect it to the wider literature.*

**Response**: Thank you for the positive feedback!

*In the introduction section it will be good to mention that*

*a) India has ratified WHO FCTC in 2004, which has specific articles (especially 9 and 10) that aim to reduce the impact of SLT use. And it has exceeded the recommended provisions and enforced a ban considering the health impact of SLTs.*

*b) That this ban was a result of multisectoral coordination between NGOs, Supreme Court and policy makers.*

*Here is a WHO source for reference: (p13) and please use other sources as needed* [*https://apps.who.int/iris/bitstream/handle/10665/363378/9789240052291-eng.pdf*](https://apps.who.int/iris/bitstream/handle/10665/363378/9789240052291-eng.pdf) *Mentioning this background info will connect this article to wider FCTC literature/be easily discoverable in searches and provide better context.*

**Response**: We agree with the reviewer’s assessment. We have made the changes and added the references. The revised text reads as follows:

“Considering the detrimental health impact of SLTs, India enacted a number of international and domestic tobacco control laws to discourage consumption. In 2004, it ratified the WHO Framework Convention on Tobacco Control (WHO-FCTC), and since then, several best practices have been implemented [8]. More recently, several state governments in India used food laws and banned the sale of gutka and pan masala—popular SLT variants—as a result of multi-sectoral coordination between the Supreme court, policymakers, and NGOs [9,10].” (Line nos: 50-56, Page no: 3)

*In the methods section: Mention the location (state/country) from where the search was conducted in Google – as Google search results are impacted by location. The fact that you conducted this search from India will be a strength.*

**Response**: As suggested by the reviewer, we have included the information in the search strategy. The text reads as follows:

“The searches were carried out in Bengaluru, the city capital of Karnataka state in southern India.” (Line nos 97-98, Page no: 4)

*Discussion: Suggest including a sentence or two about other countries that have banned SLT in the region as you speak about LMICs maybe around line 410. Line 409: Suggest rephrasing to something like “As combating the use of SLT is becoming a policy priority” the current wording is a little unclear.*

**Response**: Thank you for the feedback. We have made the suggested changes. The revised text reads as follows:

“Combating the use of SLT is slowly but surely becoming a policy priority in Southeast Asia, with countries such as Nepal, Bhutan, and Bangladesh amending existing tobacco control laws to prohibit SLT in public places, ban manufacturing and supply, and advertising. Simultaneously, multi-pronged strategies such as cessation services, pictorial warnings, taxation, education and awareness, vendor licensing, and litigation measures are being implemented [31,51,52]” (Line nos: 384-389, Page no: 21)

*Line 415: I think it is good to say WHO FCTC than WHO – as they provide guidance on supply reduction etc. and is separate from WHO*

**Response**: We have made the necessary corrections. The revised text reads as:

“Further investigation into the illicit supply-chain routes, enforcement timings, and complex social networks can empower tobacco control advocates and implementers in devising evidence-based interventions to ensure that the WHO-FCTC supply reduction measures are effectively implemented.” (Line nos: 390-393, Page no: 21)
